# Supplementary material for: Diverse enteric bacterial, viral, and parasitic pathogen genes are shed in animal feces in Indiana
Source: PLoS One. 2026 Feb 6;21(2):e0335338. doi: 10.1371/journal.pone.0335338 (PMC12880659; doi:10.1371/journal.pone.0335338)
Supplement: S1 Table — Estimates reflect (i) annual fecal mass per individual animal or person, (ii) total state inventory/population, and (iii) calculated annual fecal mass produced (kg/year). (PDF) [file pone.0335338.s001.pdf]

S1 Table. Estimated annual fecal mass production by Indiana livestock and humans, based on 2022 inventory/population data and standard manure production coefficients (Indiana, USA).

Population group	Population (2022)	Daily excretion (kg animal^{-1} d^{-1})†	Annual manure load (kg yr^{-1})	Reference
Broiler chicken	8.20×10^{6}	0.102	3.05×10^{8}	(1,2)
Layer hen	3.60×10^{7}	0.088	1.16×10^{9}	(1,2)
Turkey	8.50×10^{6}	0.184	5.71×10^{8}	(1,2)
Hog / pig	4.40×10^{6}	3.8	6.10×10^{9}	(1,2)
Cattle & calves	7.70×10^{5}	38	1.07×10^{10}	(1,2)
Human	6.80×10^{6}	0.128	3.18×10^{8}	(2,3)

Estimates reflect (i) annual fecal mass per individual animal or person, (ii) total state inventory/population, and (iii) calculated annual fecal mass produced (kg/year).

†Wet (as-excreted) manure, not dry solids. Broiler excretion factor calculated as 4.9 kg finished-bird ÷ 48 d finishing period (Table 1.a); turkey factor is the mean of male and female values [(36 kg ÷ 133 d) + (17 kg ÷ 105 d)]/2 (Table 1.a); swine factor corresponds to a 200 kg boar (Table 1.b); cattle factor to a dry dairy cow (Table 1.b).
