# Supplementary material for: Diverse enteric bacterial, viral, and parasitic pathogen genes are shed in animal feces in Indiana
Source: PLoS One. 2026 Feb 6;21(2):e0335338. doi: 10.1371/journal.pone.0335338 (PMC12880659; doi:10.1371/journal.pone.0335338)
Supplement: S2 Table — Table summarizes stool input mass (mg), double stranded DNA (dsDNA) concentration/yield (Qubit), and the calculated equivalent stool mass represented in each TaqMan Array Card (TAC) reaction following dilution and loading procedures. (PDF) [file pone.0335338.s002.pdf]

S2 Table. Nucleic-acid extraction and template input characteristics for fecal samples collected from 10 host species at 10 sites in southern Indiana, April–June 2024.

Species	Sample ID	Stool input mass (mg)	[dsDNA] (ng/µL)	Equivalent Sample Mass (mg)
Cat	Cat 1	80.6	42.0	3.224
	Cat 2	101.4	31.8	4.056
	Cat 3	45.1	41.8	1.804
	Cat 4	94.2	44	3.768
	Cat 5	118.8	24.7	4.752
	Cat 6	87.1	11.4	3.484
	Cat 7	124.1	6.81	4.964
	Cat 8	95.6	18.6	3.824
	Cat 9	170.2	17.7	6.808
	Cat 10	98.3	40.9	3.932
	Cat 11	123.1	48.5	4.924
	Cat 11 duplicate	92.5	41.5	3.7
	Cat 12	137.4	32.6	5.496
	Cat 12 duplicate	62.1	18.0	2.484
Chicken	Chicken 1	30.7	0.11	1.228
	Chicken 2	53.4	2.1	2.136
	Chicken 3	90.7	3.29	3.628
	Chicken 4	40.9	7.26	1.636
	Chicken 5	33.3	9.33	1.332
	Chicken 6	94.9	8.4	3.796
	Chicken 6 duplicate	69.2	4.36	2.768
	Chicken 7	26.3	4.64	1.052

	Chicken 7 duplicate	21.5	4.63	0.86
	Chicken 8	101.8	7.88	4.072
	Chicken 8 duplicate	103.3	5.59	4.132
	Chicken 9	54.7	2.14	2.188
	Chicken 10	37.7	14.5	1.508
	Chicken 11	96	0.123	3.84
	Chicken 12	60.4	0.15	2.416
Cow	Cow 1	49.8	3.97	1.992
	Cow 2	120.8	12.4	4.832
	Cow 2 duplicate	117.6	13.0	4.704
	Cow 3	129.6	9.48	5.184
	Cow 3 duplicate	119.1	8.35	4.764
	Cow 4	94.3	12.9	3.772
	Cow 4 duplicate	106.8	14.5	4.272
	Cow 5	68.6	6.21	2.744
	Cow 6	153.4	8.8	6.136
	Cow 7	126.2	18.0	5.048
	Cow 8	121	10.3	4.84
	Cow 9	103.8	6.71	4.152
	Cow 10	86.6	8.31	3.464
	Cow 11	115.8	10.3	4.632
	Cow 12	100.7	13.0	4.028
Deer	Deer 1	51.6	15.3	2.064

	Deer 1 duplicate	71.3	16.3	2.852
	Deer 2	52.7	4.34	2.108
	Deer 3	83.6	3.18	3.344
	Deer 4	123.1	21.8	4.924
	Deer 5	63.4	10.4	2.536
	Deer 6	74.7	15.3	2.988
	Deer 7	7.1	2.94	0.284
	Deer 7 duplicate	5.9	4.92	0.236
	Deer 8	14.7	8.69	0.588
	Deer 9	39.9	23.8	1.596
	Deer 9 duplicate	48.5	37.8	1.94
	Deer 10	41.5	31.4	1.66
	Deer 10 duplicate	40.6	24.8	1.624
	Deer 11	37.4	41.4	1.496
	Deer 12	70.4	too high	2.816
Dog	Dog 1	28.5	9.83	1.14
	Dog 2	83.9	14.3	3.356
	Dog 3	42.3	8.76	1.692
	Dog 3 duplicate	79.4	8.73	3.176
	Dog 4	31.8	29.8	1.272
	Dog 5	29.4	6.86	1.176
	Dog 5 duplicate	63.7	22.9	2.548
	Dog 6	81.1	5.8	3.244

	Dog 7	55.2	25.4	2.208
	Dog 8	51.1	7.57	2.044
	Dog 9	65.3	43.6	2.612
	Dog 10	57.7	14.2	2.308
	Dog 11	49.5	too low	1.98
	Dog 12	70.9	40.7	2.836
	Dog 12 duplicate	72.9	44.7	2.916
	Dog 13	55.3	39.1	2.212
	Dog 14	119.5	14.2	4.78
	Dog 15	76.0	35.0	3.04
	Dog 16	51.3	42.1	2.052
	Dog 17	94.9	29.7	3.796
	Dog 18	110.6	53	4.424
	Dog 19	85.8	37.9	3.432
	Dog 20	75.3	37.2	3.012
	Dog 21	49.1	6.25	1.964
	Dog 21 duplicate	24.3	3.46	0.972
	Dog 22	80.0	44.7	3.2
	Dog 22 duplicate	93.5	12.4	3.74
Goat	Goat 1	47.4	36.8	1.896
	Goat 2	54.0	36.5	2.16
	Goat 2 duplicate	45.2	34.5	1.808
	Goat 3	81.3	33.6	3.252
	Goat 3 duplicate	72.5	28.2	2.9

	Goat 4	120.5	too low	4.82
	Goat 4 duplicate	66.9	26.4	2.676
	Goat 5	56.4	39.6	2.256
	Goat 6	25.3	27.8	1.012
	Goat 7	40.2	29.3	1.608
	Goat 8	32.4	34.9	1.296
	Goat 9	58.6	27.1	2.344
	Goat 10	79.6	23.9	3.184
	Goat 11	50.8	31.8	2.032
	Goat 12	87.5	28.6	3.5
Horse	Horse 1	43.3	15.1	1.732
	Horse 2	68.7	too high	2.748
	Horse 3	54.5	7.86	2.18
	Horse 4	69.7	20.2	2.788
	Horse 5	29.6	27.1	1.184
	Horse 6	45.1	10.7	1.804
	Horse 7	48.3	15.4	1.932
	Horse 8	63.5	9.93	2.54
	Horse 9	55.3	13.4	2.212
	Horse 10	108.1	28.7	4.324
	Horse 11	54.3	41.5	2.172
	Horse 11 duplicate	36.4	10.6	1.456
	Horse 12	104.2	24.9	4.168
	Horse 12 duplicate	45.0	25.2	1.8
Human	Human 1	24.2	too high	0.968

	Human 2	64.1	too high	2.564
	Human 3	70.8	too high	2.832
	Human 4	72.0	too high	2.88
	Human 5	31.6	too high	1.264
	Human 5 duplicate	71.6	too high	2.864
	Human 6	19.1	too high	0.764
	Human 7	64.2	too high	2.568
	Human 8	71.1	too high	2.844
	Human 9	59.0	too high	2.36
	Human 10	115.7	too high	4.628
Pig	Pig 1	74.8	10.6	2.992
	Pig 2	106.5	16.1	4.26
	Pig 3	76.3	13.7	3.052
	Pig 4	90	15.7	3.6
	Pig 5	108.6	12.8	4.344
	Pig 6	70.5	18.9	2.82
	Pig 7	78.9	14.0	3.156
	Pig 8	19.6	8.97	0.784
	Pig 8 duplicate	22.2	10.4	0.888
	Pig 9	69.3	12.2	2.772
	Pig 9 duplicate	42.1	10.8	1.684
	Pig 10	54.8	15.1	2.192
	Pig 11	46.6	10.5	1.864
	Pig 12	43.5	20.7	1.74
	Pig 12 duplicate	42.3	17.5	1.692
Sheep	Sheep 1	40.6	21.6	1.624

	Sheep 2	46.9	20.4	1.876
	Sheep 3	35.9	29.9	1.436
	Sheep 4	38.6	27.3	1.544
	Sheep 5	20.6	8.67	0.824
	Sheep 6	39.5	20.9	1.58
	Sheep 7	45.9	17.6	1.836
	Sheep 8	37.1	21.4	1.484
	Sheep 8 duplicate	33.6	14.8	1.344
	Sheep 9	39.0	16.7	1.56
	Sheep 9 duplicate	29.6	17.7	1.184
	Sheep 10	29.6	12.7	1.184
	Sheep 11	19.7	19.4	0.788
	Sheep 12	20.2	19.8	0.808
	Sheep 12 duplicate	16.3	19.0	0.652

Table summarizes stool input mass (mg), double stranded DNA (dsDNA) concentration/yield (Qubit), and the calculated equivalent stool mass represented in each TaqMan Array Card (TAC) reaction following dilution and loading procedures.
