# Supplementary material for: Diverse enteric bacterial, viral, and parasitic pathogen genes are shed in animal feces in Indiana
Source: PLoS One. 2026 Feb 6;21(2):e0335338. doi: 10.1371/journal.pone.0335338 (PMC12880659; doi:10.1371/journal.pone.0335338)
Supplement: S3 Table — For each assay, the table lists target organism/marker, gene/region, and oligonucleotide sequences (5′ → 3′) for forward primer, reverse primer, and hydrolysis probe. TAC = TaqMan Array Card. RT-qPCR = Reverse-Transcription Quantitative Polymerase Chain Reaction. (PDF) [file pone.0335338.s003.pdf]

S3 Table. Primer and probe sequences for the custom TAC RT-qPCR assays used to screen fecal samples from 10 host species collected at 10 sites in southern Indiana, April–June 2024.

Type	Target	Gene	Primer or probe sequence (5' - 3')	Reference
Virus	Adenovirus 40/41	Fiber gene	Fwd: AACTTTCTCTCTTAATAGACGCC	(4)
			Rev: AGGGGGCTAGAAAACAAAA	
			Probe: CTGACACGGGCACTCT	
	Astrovirus	Capsid	Fwd: CAGTTGCTTGCTGCGTTCA	(4)
			Rev: CTTGCTAGCCATCACACTTCT	
			Probe: CACAGAAGAGCAACTCCATCGC	
	BHV	Glycoprotein B gene	Fwd: GAGCAAAGCCCCGCCGAAGGA	(5)
			Rev: TACGAACAGCAGCACGGGCGG	
			Probe: GAACCTGCCCACGCGCTGAAAC	
	BRSV	Nucleoprotein gene	Fwd: GCAATGCTGCAGGACTAGGTATAAT	(6)
			Rev: ACACTGTAATTGATGACCCCATTCT	
			Probe: ACCAAGACTTGTATGATGCTGCCAAAGCA	
	Influenza A	Matrix	Fwd: CAAGACCAATCYTGTCACCTCTGAC	(7)
			Rev: GCATTYTGGACAAAVCGTCTACG	
			Probe: TGCAGTCCTCGCTCACTGGGCACG	
	Norovirus GI	ORF1-2	Fwd: CGYTGGATGCGNTTYCATGA	(4)
			Rev: CTTAGACGCCATCATCATTYAC	
			Probe: TGGACAGGAGATCGC	

	Norovirus GII	ORF1-2	Fwd: CARGARBCNATGTTYAGRTGGATGAG	(4)
			Rev: TCGACGCCATCTTCATTCACA	
			Probe: TGGGAGGGCGATCGCAATCT	
	RSV	N gene of RSV A & RSV B genome	Fwd: CTCCAGAATAYAGGCATGAYTCTCC	(8)
			Rev: GCYCTYCTAATYACWGCTGTAAGAC	
			Probe: TAACCAAATTAGCAGCAGGAGATAGATCAG	
	Rotavirus	NSP3	Fwd: ACCATCTWCACRTRACCCTCTATGAG	(4)
			Rev: GGTCACATAACGCCCCTATAGC	
			Probe: AGTTAAAAGCTAACACTGTCAAA	
	SARS-CoV-2	Nucleocapsid	Fwd: CTGCAGATTTGGATGATTTCTCC	(7)
			Rev: CCTTGTGTGGTCTGCATGAGTTTAG	
			Probe: ATTGCAACAATCCATGAGCAGTGCTGACTC	
	Sapovirus	RdRp	Fwd: GAYCASGCTCTCGCYACCTAC	(4)
			Rev: CCCTCCATYTCAAACACTA	
			Probe: GAYCASGCTCTCGCYACCTAC	
Bacteria	*Campylobacter jejuni/coli*	*cadF*	Fwd: CTGCTAAACCATAGAAATAAAATTTCTCAC	(4)
			Rev: CTTTGAAGGTAATTTAGATATGGATAATCG	

			Probe: CATTTTGACGATTTTTGGCTTGA	
	Clostridioides difficile	*tcdB*	Fwd: GGTATTACCTAATGCTCCAAATAG	(4)
			Rev: TTTGTGCCATCATTTTCTAAGC	
			Probe: CCTGGTGTCCATCCTGTTTC	
	Enteroaggregative *E. coli* (aaiC)	aaiC	Fwd: ATTGTCCTCAGGCATTTCAC	(4)
			Rev: ACGACACCCCTGATAAACAA	
			Probe: TAGTGCATACTCATCATTTAAG	
	Enteroaggregative *E. coli* (aatA)	aatA	Fwd: CTGGCGAAAGACTGTATCAT	(4)
			Rev: TTTTGCTTCATAAGCCGATAGA	
			Probe: TGGTTCTCATCTATTACAGACAGC	
	Enteropathogenic *E. coli* (bfpA)	bfpA	Fwd: TGGTGCTTGCGCTTGCT	(4)
			Rev: CGTTGCGCTCATTACTTCTG	
			Probe: CAGTCTGCGTCTGATTCCAA	
	Enteropathogenic *E. coli* (eae)	eae	Fwd: CATTGATCAGGATTTTTCTGGTGATA	(4)
			Rev: CTCATGCGGAAATAGCCGTTA	
			Probe: ATACTGGCGAGACTATTTCAA	
	Enterotoxigenic *E. coli* (LT)	LT	Fwd: TTCCCACCGGATCACCAA	(4)
			Rev: CAACCTTGTGGTGCATGATGA	
			Probe: CTTGGAGAGAAGAACCCT	

	Enterotoxigenic *E. coli* (STh)	STh	Fwd: GCTAAACCAGYAGRGTCTTCAAAA	(4)
			Rev: CCCGGTACARGCAGGATTACAACA	
			Probe: TGGTCCTGAAAGCATGAA	
	Enterotoxigenic *E. coli* (STp)	STp	Fwd: TGAATCACTTGACTCTTCAAAA	(4)
			Rev: GGCAGGATTACAACAAAGTT	
			Probe: TGAACAACACATTTTACTGCT	
	Escherichia coli O157:H7	*rfbE*	Fwd: TTTCACACTTATTGGATGGTCTCAA	(4)
			Rev: CGATGAGTTTATCTGCAAGGTGAT	
			Probe: CTCTCTTTCCTCTGCGGTCCT	
	Helicobacter pylori	*ureC*	Fwd: GACACCAGAAAAAGCGGCTA	(4)
			Rev: AGCGCATGTCTTCGGTTAAA	
			Probe: TCACTAAAGCGTTTTCTACC	
	Klebsiella pneumoniae	Diguanylate cyclase	Fwd: TGCAGATAATTCACGCCCAG	(9)
			Rev: ACCCGCTGGACGCCAT	
			Probe: CCACCACGCTCATCTGTTTCGCC	
	Plesiomonas shigelloides	*gyrB*	Fwd: CCGCCGTGAAGGCAAAG	(4)
			Rev: GCTACCGGCTCACCCAGAT	
			Probe: CACACCCAAGAATAC	
	Salmonella enterica serovar Typhi	STY0201	Fwd: CGCGAAGTCAGAGTCGACATAG	(4)
			Rev: AAGACCTCAACGCCGATCAC	
			Probe: CAGCCTGCTCCAGAACA	

	Salmonella spp.	*ttr*	Fwd: CTCACCAGGAGATTACAACATGG	(4)
			Rev: AGCTCAGACCAAAAGTGACCATC	
			Probe: CACCGACGGCGAGACCGACTTT	
	Shiga-toxin producing *E. coli* (*stx1*)	*stx1*	Fwd: ACTTCTCGACTGCAAAGACGTATG	(4)
			Rev: ACAAATTATCCCCTGWGCCACTATC	
			Probe: CTCTGCAATAGGTACTCCA	
	Shiga-toxin producing *E. coli* (*stx2*)	*stx2*	Fwd: CCACATCGGTGTCTGTTATTAACC	(4)
			Rev: GGTCAAAACGCGCCTGATAG	
			Probe: TTGCTGTGGATATACGAGG	
	Shigella / EIEIC	*ipaH*	Fwd: CCTTTTCCGCGTTCCTTGA	(4)
			Rev: CGGAATCCGGAGGTATTGC	
			Probe: CGCCTTTCCGATACCGTCTCTGCA	
	Vibrio cholerae	*hlyA*	Fwd: GTTTGGCGAGAGCAAGGTTT	(4)
			Rev: TCTCTTCTTCAACCGTTTCCA	
			Probe: CGCAGAGTCGAAATGGCTTGG	
	Yersinia enterocolitica	*lytA*	Fwd: TGATTCACCAGCAGCAATAC	(4)
			Rev: GGCATCATGAAAGGCGG	
			Probe: TGTCGGTTTCTCCTTCCAGG	
Fungi	*Candida auris*	ITS2 region of Ribosome gene	Fwd: CAGACGTGAATCATCGAATCT	(10)
			Rev: TTTCGTGCAAGCTGTAATTT	

			Probe: AATCTTCGCGGTGGCGTTGCATTCA	
Protozoa	*Cryptosporidium* spp.	18S rRNA	Fwd: GGGTTGTATTTATTAGATAAAGAACCA	(4)
			Rev: AGGCCAATACCCTACCGTCT	
			Probe: TGACATATCATTCAAGTTTCTGAC	
	Entamoeba histolytica	18S rRNA	Fwd: ATTGTCGTGGCATCCTAACTCA	(4)
			Rev: GCGGACGGCTCATTATAACA	
			Probe: TCATTGAATGAATTGGCCATTT	
	Giardia spp.	18S rRNA	Fwd: GACGGCTCAGGACAACGGTT	(4)
			Rev: TTGCCAGCGGTGTCCG	
			Probe: CCCGCGGCGGTCCCTGCTAG	
	Plasmodium spp.	18S rRNA	Fwd: GCTCTTTCTTGATTTCTTGGATG	(11)
			Rev: AGCAGGTTAAGATCTCGTTCG	
			Probe: CACGAACTAAAAACGGCCAT	
Helminth	*Ancylostoma duodenale*	*ITS2*	Fwd: GAATGACAGCAAACTCGTTGTTG	(4)
			Rev: ATACTAGCCACTGCCGAAACGT	
			Probe: ATCGTTTACCGACTTTAG	
	Ascaris lumbricoides	*ITS1*	Fwd: GCCACATAGTAAATTGCACACAAAT	(4)
			Rev: GCCTTTCTAACAAGCCCAACAT	
			Probe: TTGGCGGACAATTGCATGCGAT	
	Necator americanus	*ITS2*	Fwd: CTGTTTGTCGAACGGTACTTGC	(4)
			Rev: ATAACAGCGTGCACATGTTGC	

			Probe: CTGTACTACGCATTGTATAC	
	Shistosoma mansoni	*S.mansoni* mitochondrion	Fwd: GGTCTAGATGACTTGATYGAGATGCT	(12)
			Rev: TCCCGAGCGYGTATAATGTCATTA	
			Probe: TGGGTTGTGCTCGAGTCGTGGC	
	Strongyloides stercoralis	Dispersed repetitive sequence	Fwd: TCCAGAAAAGTCTTCACTCTCCAG	(4)
			Rev: TGCGTTAGAATTTAGATATTATTGTTGCT	
			Probe: TCAGCTCCAGTTGAACAACAGCCTCCAA	
	Trichuris trichiura	18S rRNA	Fwd: TTGAAACGACTTGCTCATCAACTT	(4)
			Rev: CTGATTCTCCGTTAACCGTTGTC	
			Probe: CGATGGTACGCTACGTGCTTACCATGG	
Control	16S rRNA	16S rRNA	Fwd: ATGGYTGTCGTCAGCT	(13)
			Rev: ACGGGCGGTGTGTAC	
			Probe: CAACGAGCGCAACCC	
	Class 1 Resistance Integron (RI)	*intl1*	Fwd: GATCGGTCGAATGCGTGT	(14)
			Rev: GCCTTGATGTTACCCGAGAG	
			Probe: ATTCCTGGCCGTGGTTCTGGGTTTT	
	Human mtDNA	Cytochrome b gene of the human mitochondrial genome	Fwd: CAATGAATCTGAGGAGGCTAC	(15)

			Rev: CGTGCAAGAATAGGAGGTG	
			Probe: ACCCTCACACGATTCTTTACCTTTCACT	
	Leukocyte	*RHCE*	Fwd: TGGCCACCATGAGTGCTATGT	(16)
			Rev: CTCCACCAGCACCATCACC	
			Probe: GATCTCAGCGGGTGCT	

For each assay, the table lists target organism/marker, gene/region, and oligonucleotide sequences (5′→3′) for forward primer, reverse primer, and hydrolysis probe. TAC = TaqMan Array Card. RT-qPCR = Reverse-Transcription Quantitative Polymerase Chain Reaction.

References

1. Manure Production and Characteristics. St. Joseph, MI, USA: American Society of Agricultural Engineers; 2005 Mar p. 20. (ASAE Standards). Report No.: ASAE D384.2 MAR2005.

2. 2022 Census of Agriculture: Indiana State and County Data [Internet]. 2022. Available from: https://www.nass.usda.gov/Publications/AgCensus/2022/Full_Report/Volume_1,_Chapter_1_State_Level/Indiana/

3. Rose C, Parker A, Jefferson B, Cartmell E. The Characterization of Feces and Urine: A Review of the Literature to Inform Advanced Treatment Technology. Crit Rev Environ Sci Technol. 2015 Sep 2;45(17):1827–79.

4. Liu J, Gratz J, Amour C, Nshama R, Walongo T, Maro A, et al. Optimization of Quantitative PCR Methods for Enteropathogen Detection. Chan KH, editor. PLOS ONE. 2016 Jun 23;11(6):e0158199.

5. Wang J, O'Keefe J, Orr D, Loth L, Banks M, Wakeley P, et al. Validation of a real-time PCR assay for the detection of bovine herpesvirus 1 in bovine semen. J Virol Methods. 2007 Sep;144(1–2):103–8.

6. Boxus M, Letellier C, Kerkhofs P. Real Time RT-PCR for the detection and quantitation of bovine respiratory syncytial virus. J Virol Methods. 2005 May;125(2):125–30.

7. CDC's Influenza SARS-CoV-2 Multiplex Assay [Internet]. U.S. Centers for Disease Control and Prevention; 2022 Nov. Available from: https://archive.cdc.gov/#/details?url=https://www.cdc.gov/coronavirus/2019-ncov/lab/multiplex.html

8. Hughes B, Duong D, White BJ, Wigginton KR, Chan EMG, Wolfe MK, et al. Respiratory Syncytial Virus (RSV) RNA in Wastewater Settled Solids Reflects RSV Clinical Positivity Rates. Environ Sci Technol Lett. 2022 Feb 8;9(2):173–8.

9. Diaz MH, Waller JL, Napoliello RA, Islam MdS, Wolff BJ, Burken DJ, et al. Optimization of Multiple Pathogen Detection Using the TaqMan Array Card: Application for a Population-Based Study of Neonatal Infection. Lin B, editor. PLoS ONE. 2013 Jun 21;8(6):e66183.

10. Leach L, Zhu Y, Chaturvedi S. Development and Validation of a Real-Time PCR Assay for Rapid Detection of Candida auris from Surveillance Samples. Warnock DW, editor. J Clin Microbiol [Internet]. 2018 Feb [cited 2025 Jul 23];56(2). Available from: https://journals.asm.org/doi/10.1128/JCM.01223-17

11. Liu J, Ochieng C, Wiersma S, Ströher U, Towner JS, Whitmer S, et al. Development of a TaqMan Array Card for Acute-Febrile-Illness Outbreak Investigation and Surveillance of Emerging Pathogens, Including Ebola Virus. McAdam AJ, editor. J Clin Microbiol. 2016 Jan;54(1):49–58.

12. Ten Hove RJ, Verweij JJ, Vereecken K, Polman K, Dieye L, Van Lieshout L. Multiplex real-time PCR for the detection and quantification of Schistosoma mansoni and S. haematobium infection in stool samples collected in northern Senegal. Trans R Soc Trop Med Hyg. 2008 Feb;102(2):179–85.

13. Ritalahti KM, Amos BK, Sung Y, Wu Q, Koenigsberg SS, Löffler FE. Quantitative PCR Targeting 16S rRNA and Reductive Dehalogenase Genes Simultaneously Monitors Multiple*Dehalococcoides*Strains. Appl Environ Microbiol. 2006 Apr;72(4):2765–74.

14. Barraud O, Baclet MC, Denis F, Ploy MC. Quantitative multiplex real-time PCR for detecting class 1, 2 and 3 integrons. J Antimicrob Chemother. 2010 Aug 1;65(8):1642–5.

15. Zhu K, Suttner B, Pickering A, Konstantinidis KT, Brown J. A novel droplet digital PCR human mtDNA assay for fecal source tracking. Water Res. 2020 Sep;183:116085.

16. Doescher A, Loges U, Petershofen EK, Müller TH. Evaluation of droplet digital PCR for quantification of residual leucocytes in red blood cell concentrates. Vox Sang. 2017 Nov;112(8):744–50.

17. Capone D, Berendes D, Cumming O, Knee J, Nalá R, Risk BB, et al. Analysis of Fecal Sludges Reveals Common Enteric Pathogens in Urban Maputo, Mozambique. Environ Sci Technol Lett. 2020 Dec 8;7(12):889–95.

18. Capone D, Bakare T, Barker T, Chatham AH, Clark R, Copperthwaite L, et al. Risk Factors for Enteric Pathogen Exposure among Children in Black Belt Region of Alabama, USA. Emerg Infect Dis [Internet]. 2023 Dec [cited 2025 Jul 22];29(12). Available from: https://wwwnc.cdc.gov/eid/article/29/12/23-0780_article

19. Liu J, Gratz J, Amour C, Kibiki G, Becker S, Janaki L, et al. A Laboratory-Developed TaqMan Array Card for Simultaneous Detection of 19 Enteropathogens. J Clin Microbiol. 2013 Feb;51(2):472–80.

20. Rousselon N, Delgenès JP, Godon JJ. A new real time PCR (TaqMan® PCR) system for detection of the16S rDNA gene associated with fecal bacteria. J Microbiol Methods. 2004 Oct;59(1):15–22.
