# Supplementary material for: Diverse enteric bacterial, viral, and parasitic pathogen genes are shed in animal feces in Indiana
Source: PLoS One. 2026 Feb 6;21(2):e0335338. doi: 10.1371/journal.pone.0335338 (PMC12880659; doi:10.1371/journal.pone.0335338)
Supplement: S4 Table — Table reports slope, y-intercept, R², and calculated amplification efficiency for each assay based on an eight-point 10-fold dilution series. Limit of detection (LOD) for assay validation was 100 gene copies/µL. TAC = TaqMan Array Card. (PDF) [file pone.0335338.s004.pdf]

S4 Table. Standard-curve performance characteristics for assays included on the custom TAC RT-qPCR panel used to detect enteric microbial and parasitic nucleic-acid targets in fecal samples from southern Indiana, April–June 2024.

Type	Target	Target Gene	R^2	Slope	Efficiency	Y-intercept
Virus	Astrovirus	Capsid	0.996	-3.1455	108%	38.672
	BHV	Glycoprotein B gene	0.996	-3.0444	113%	35.118
	BRSV	Nucleoprotein gene	0.999	-3.0942	110%	36.838
	Influenza A	Matrix	0.993	-2.9178	120%	37.548
	Norovirus GI	ORF1-2	0.995	-3.1883	106%	37.887
	Norovirus GII	ORF1-2	0.998	-3.1862	106%	39.006
	RSV	N gene of RSV A & RSV B genome	0.993	-3.216	105%	38.591
	Rotavirus	NSP3	0.998	-3.2407	104%	40.576
	SARS-CoV-2	Nucleocapsid	0.997	-3.0719	112%	36.899
	Sapovirus	RdRp	0.997	-2.9557	118%	37.623
Bacteria	*Campylobacter jejuni/coli*	*cadF*	0.999	-3.2126	105%	37.353
	Clostridioides difficile	*tcdB*	0.999	-3.2299	104%	36.817
	Enteroaggregative *E. coli* (aaiC)	aaiC	0.999	-3.2254	104%	36.075
	Enteroaggregative *E. coli* (aatA)	aatA	0.998	-3.1901	106%	35.434
	Enteropathogenic *E. coli* (bfpA)	bfpA	0.999	-3.1463	108%	35.91
	Enteropathogenic *E. coli* (eae)	eae	0.999	-3.1697	107%	36.966
	Enterotoxigenic *E. coli* (LT)	LT	0.999	-3.2241	104%	36.234
	Enterotoxigenic *E. coli* (STh)	STh	0.999	-3.2538	103%	37.891
	Enterotoxigenic *E. coli* (STp)	STp	0.998	-3.1793	106%	36.16
	Escherichia coli O157:H7	*rfbE*	0.999	-3.1794	106%	36.154
	Helicobacter pylori	*ureC*	0.997	-3.2083	105%	36.828
	Klebsiella pneumoniae	Diguanylate cyclase	0.997	-3.177	106%	35.882
	Plesiomonas shigelloides	*gyrB*	0.998	-3.1965	106%	36.393

	Salmonella enterica serovar Typhi	STY0201	0.999	-3.2506	103%	37.968
	Salmonella spp.	*ttr*	0.997	-3.3111	100%	36.402
	Shiga-toxin producing *E. coli* (stx1)	*stx1*	0.999	-3.2542	103%	38.4949
	Shiga-toxin producing *E. coli* (stx2)	*stx2*	0.999	-3.2254	104%	37.95
	Shigella / EIEIC	*ipaH*	0.999	-3.1979	105%	37.014
	Vibrio cholerae	*hlyA*	0.999	-3.2278	104%	37.318
	Yersinia enterocolitica	*lytA*	0.997	-3.2678	102%	37.094
Fungi	*Candida auris*	ITS2 region of Ribosome gene	0.996	-3.0974	110%	35.079
Protozoa	*Cryptosporidium* spp.	18S rRNA	0.997	-3.1954	106%	36.527
	Entamoeba histolytica	18S rRNA	0.998	-3.3706	98%	38.097
	Giardia spp.	18S rRNA	0.999	-3.2343	104%	36.52
	Plasmodium spp.	18S rRNA	0.999	-3.184	106%	36.832
Helminth	*Ancylostoma duodenale*	*ITS2*	0.999	-3.2636	102%	37.788
	Ascaris lumbricoides	*ITS1*	0.999	-3.1673	107%	36.312
	Necator americanus	*ITS2*	0.997	-3.2457	103%	39.44
	Shistosoma mansoni	*S.mansoni* mitochondrion	0.999	-3.191	106%	36.398
	Strongyloides stercoralis	Dispersed repetitive sequence	0.998	-3.109	110%	36.52
	Trichuris trichiura	18S rRNA	0.999	-3.1886	106%	36.88
Control	16S rRNA	16S rRNA	0.998	-3.1537	108%	35.397
	Class 1 Resistance Integron (RI)	*intl1*	0.996	-3.2191	104%	36.378
	Human mtDNA	Cytochrome b gene of the human mitochondrial	0.992	-3.0189	114%	34.087

		genome				

Table reports slope, y-intercept, R^2, and calculated amplification efficiency for each assay based on an eight-point 10-fold dilution series. Limit of detection (LOD) for assay validation was 100 gene copies/μL. TAC = TaqMan Array Card.
