# Supplementary material for: Diverse enteric bacterial, viral, and parasitic pathogen genes are shed in animal feces in Indiana
Source: PLoS One. 2026 Feb 6;21(2):e0335338. doi: 10.1371/journal.pone.0335338 (PMC12880659; doi:10.1371/journal.pone.0335338)

S1 Fig. Representative amplification and multicomponent plots from custom TAC RT-qPCR runs used to detect enteric microbial and parasitic targets in fecal samples collected across 10 sites in southern Indiana, April–June 2024. Plots illustrate typical positive and negative amplification patterns used for target calling in QuantStudio analysis. TAC = TaqMan Array Card. Cq = quantification cycle. RT-qPCR = Reverse-Transcription Quantitative Polymerase Chain Reaction.

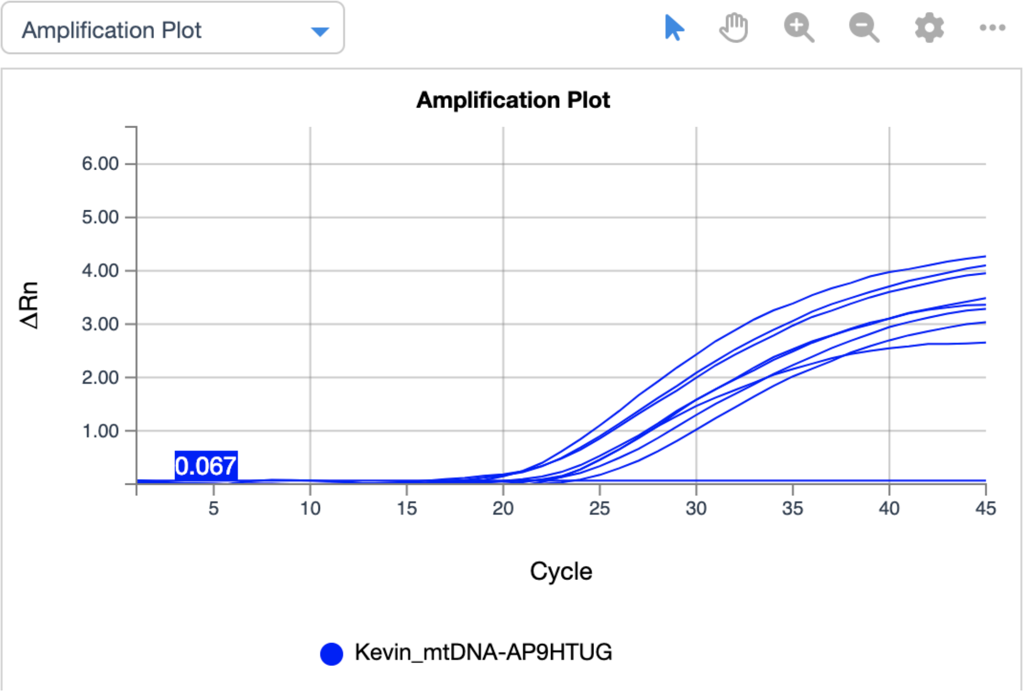

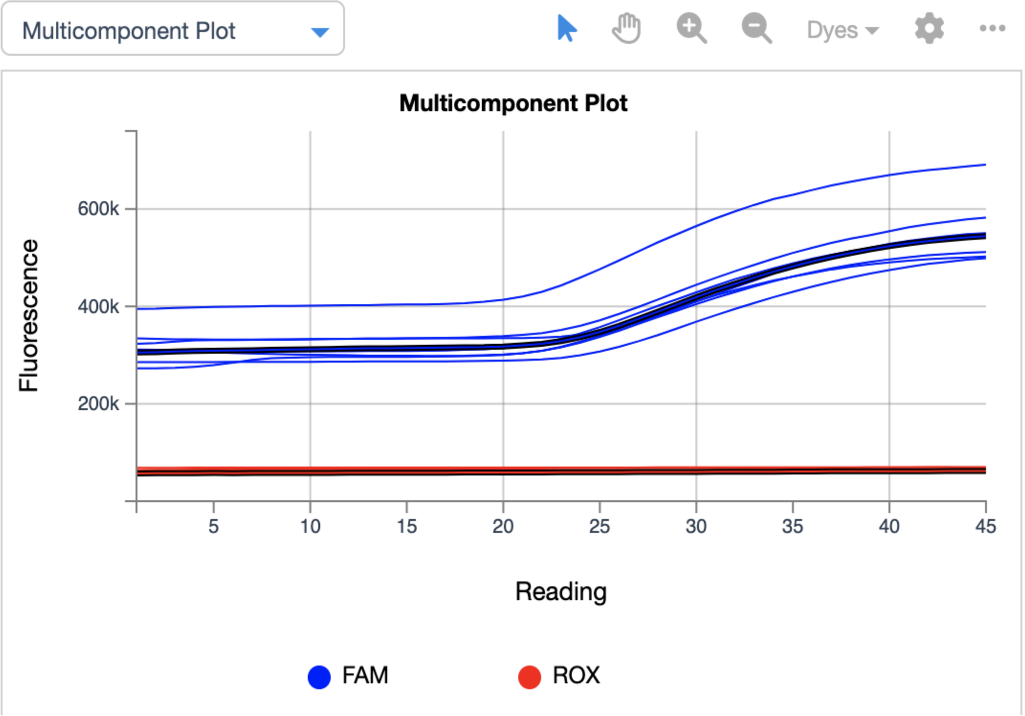

Supplement: S1 Fig — Plots illustrate typical positive and negative amplification patterns used for target calling in QuantStudio analysis. TAC = TaqMan Array Card. Cq = quantification cycle. RT-qPCR = Reverse-Transcription Quantitative Polymerase Chain Reaction. (PDF) [file pone.0335338.s005.pdf]
