# Supplementary material for: Diverse enteric bacterial, viral, and parasitic pathogen genes are shed in animal feces in Indiana
Source: PLoS One. 2026 Feb 6;21(2):e0335338. doi: 10.1371/journal.pone.0335338 (PMC12880659; doi:10.1371/journal.pone.0335338)
Supplement: S5 Table — Checklist items indicate where required methodological and reporting elements are addressed in the manuscript and supporting information. MIQE = Minimum Information for Publication of Quantitative Real-Time PCR Experiments. TAC = TaqMan Array Card. RT-qPCR = Reverse-Transcription Quantitative Polymerase Chain Reaction. (PDF) [file pone.0335338.s006.pdf]

**S5 Table. MIQE checklist for the custom TAC RT-qPCR workflow used to detect enteric microbial and parasitic targets in fecal samples collected across 10 sites in southern Indiana, April–June 2024.**

| ITEM TO CHECK | IMPORTANCE | CHECKLIST |
|---|---|---|
| **EXPERIMENTAL DESIGN** | | |
| Definition of experimental and control groups | **E** | Cross-sectional study with no intervention or control group |
| Number within each group | **E** | Cat (n=12), chicken (n=12), cow (n=12), deer (n=12), dog (n=22), goat (n=12), horse (n=12), human (n=10), pig (n=12), sheep (n=12) |
| Assay carried out by core lab or investigator's lab? | D | Investigator's lab |
| **SAMPLE** | | |
| Description | **E** | Stool samples collected across 10 sites in Southern Indiana |
| Volume/mass of sample processed | D | Median = 63.4 mg, range = 5.9 – 170.2 mg |
| Microdissection or macrodissection | **E** | Not applicable |
| Processing procedure | **E** | See methods section |
| If frozen - how and how quickly? | **E** | Stored in sterile containers at 4 ºC for <24 hours, and then frozen at -80 ºC until processing |
| If fixed - with what, how quickly? | **E** | Not fixed |
| Sample storage conditions and duration (especially for FFPE samples) | **E** | Stools samples and extracted DNA templates stored at -80 ºC |
| **NUCLEIC ACID EXTRACTION** | | |
| Procedure and/or instrumentation | **E** | See methods section |
| Name of kit and details of any modifications | **E** | QIAamp 96 Virus QIAcube HT Kit, protocol adapted from *QIAcube HT Handbook* (Qiagen, 2015) and Capone *et al.* 2020 and 2023 (17,18) |
| Source of additional reagents used | D | PowerBead Pro Tubes (Qiagen), Buffer ASL (Qiagen), Inforce 3 vaccine (Zoetis), TissueLyser II (Qiagen) |
| Details of DNase or RNAse treatment | **E** | Not applicable |
| Contamination assessment (DNA or RNA) | **E** | One negative control was included in each extraction batch |
| Nucleic acid quantification | **E** | Qubit 1X HS dsDNA Kit |

| | | |
|---|---|---|
| Instrument and method | **E** | Qubit 4 Fluorometer |
| Yield | **D** | Median = 15.55 ng/µL, range = too low – to high |
| RNA integrity method/instrument | **E** | Not measured |
| Inhibition testing (Cq dilutions, spike or other) | **E** | Monitored amplification of spiked controls |
| **qPCR TARGET INFORMATION** | | |
| If multiplex, efficiency and LOD of each assay | **E** | Table S4<br>LOD = 100 gc/µL |
| *In silico* specificity screen (BLAST, etc) | **E** | We BLASTed all assays to confirm specificity before ordering the custom TAC. |
| **qPCR OLIGONUCLEOTIDES** | | |
| Primer sequences | **E** | Table S3 |
| Probe sequences | D | Table S3 |
| Location and identity of any modifications | **E** | No modifications |
| Manufacturer of oligonucleotides | D | Thermo Fisher Scientific |
| **qPCR PROTOCOL** | | |
| Complete reaction conditions | **E** | 45°C for 20 min and 95°C for 10 min, followed by 45 cycles of 95°C for 15 sec and 60°C for 1 min |
| Reaction volume and amount of cDNA/DNA | **E** | 40 µL of 1:10 diluted extraction template, with 60 µL of AgPath-ID™ One-Step RT-PCR Reagents |
| Primer, (probe), Mg++ and dNTP concentrations | **E** | All assays contained the same concentrations of primers (900 nmol/L) and probe (250 nmol/L). The Mg2+ and dNTP concentrations are not listed in the User Guide. |
| Polymerase identity and concentration | **E** | AmpliTaq Gold™ polymerase |
| Buffer/kit identity and manufacturer | **E** | AgPath-ID™ One-Step RT-PCR Reagents |
| Additives (SYBR Green I, DMSO, etc.) | **E** | No additives |
| Manufacturer of plates/tubes and catalog number | D | Thermo Fisher Scientific |
| Complete thermocycling parameters | **E** | 45°C for 20 min and 95°C for 10 min, followed by 45 cycles of 95°C for 15 sec and 60°C for 1 min |
| Reaction setup (manual/robotic) | D | Manual set-up in a disinfected dead air box (10% bleach and 70% ethanol, followed by UV for fifteen minutes) |

| | | |
|---|---|---|
| Manufacturer of qPCR instrument | **E** | Thermo Fisher Scientific, Waltham, MA |
| **qPCR VALIDATION** | | |
| Evidence of optimisation (from gradients) | D | See Liu *et al*. 2013 (19) and Liu *et al*. 2016 (4) |
| Specificity (gel, sequence, melt, or digest) | **E** | See Liu *et al*. 2013 (19) and Liu *et al*. 2016 (4) |
| Standard curves with slope and y-intercept | **E** | Table S4 |
| PCR efficiency calculated from slope | **E** | Table S4 |
| $R^2$ of standard curve | **E** | Table S4 |
| Evidence for limit of detection | **E** | LOD established from standard curve dilution series, lowest concentration tested was 100 gene copies/µL. |
| **DATA ANALYSIS** | | |
| qPCR analysis program (source, version) | **E** | QuantStudio™ Real-Time PCR Design & Analysis Software 2.8.0 |
| Cq method determination | **E** | Manual thresholding |
| Results of NTCs | **E** | See results section |
| Justification of number and choice of reference genes | **E** | N/A |
| Software (source, version) | **E** | Python 3.8.20 (Python Software Foundation, python.org); pandas 2.0.3 (PyPI); pathlib (Python 3.8.20 standard library); Visual Studio Code 1.102.1 (Microsoft) |

Checklist items indicate where required methodological and reporting elements are addressed in the manuscript and supporting information. MIQE = Minimum Information for Publication of Quantitative Real-Time PCR Experiments. TAC = TaqMan Array Card. RT-qPCR = Reverse-Transcription Quantitative Polymerase Chain Reaction.
