# Supplementary material for: Diverse enteric bacterial, viral, and parasitic pathogen genes are shed in animal feces in Indiana
Source: PLoS One. 2026 Feb 6;21(2):e0335338. doi: 10.1371/journal.pone.0335338 (PMC12880659; doi:10.1371/journal.pone.0335338)
Supplement: S6 Table — Table includes results for all samples, including specimens later excluded from prevalence analyses due to internal-control performance, as described in the manuscript. Targets are reported as detected/not detected based on study positivity thresholds (Cq < 40 with manual thresholding). The following pathogenic targets were not detected in any samples: Ancylostoma duodenale, Necator americanus, Schistosoma mansoni, Trichuris trichiura, Entamoeba histolytica, enteroaggregative E. coli, enterotoxigenic E. coli (STh), Helicobacter pylori, Salmonella enterica serovar Typhi, Salmonella spp., Shigella spp./enteroinvasive E. coli, Vibrio cholerae, Influenza A, Norovirus GI, Rotavirus, Sapovirus, SARS-Cov2, Candida auris. TAC = TaqMan Array Card. Cq = quantification cycle. RT-qPCR = Reverse-Transcription Quantitative Polymerase Chain Reaction. (PDF) [file pone.0335338.s007.pdf]

S6 Table. Prevalence of *all* enteric microbial, parasitic, and antimicrobial-resistance gene targets detected by custom TAC RT-qPCR in *all* fecal samples from 10 host species collected at 10 sites in southern Indiana, April–June 2024.

		Prevalence (%; number positive/total samples)										
**Type**	**Target**	**Cat**	**Chicken**	**Cow**	**Deer**	**Dog**	**Goat**	**Horse**	**Pig**	**Sheep**	**Human**	**Total**
Virus	Astrovirus	0.0% (0/12)	0.0% (0/12)	0.0% (0/12)	0.0% (0/12)	0.0% (0/22)	0.0% (0/12)	8.3% (1/12)	0.0% (0/12)	0.0% (0/12)	0.0% (0/10)	0.8% (1/128)
	BHV	91.7% (11/12)	91.7% (11/12)	100.0% (12/12)	100.0% (12/12)	100.0% (22/22)	100.0% (12/12)	100.0% (12/12)	100.0% (12/12)	100.0% (12/12)	100.0% (10/10)	98.4% (126/128)
	BRSV	100.0% (12/12)	91.7% (11/12)	100.0% (12/12)	25.0% (3/12)	100.0% (22/22)	33.3% (4/12)	91.7% (11/12)	75.0% (9/12)	100.0% (12/12)	90.0% (9/10)	82.0% (105/128)
	Norovirus GII	0.0% (0/12)	0.0% (0/12)	0.0% (0/12)	0.0% (0/12)	0.0% (0/22)	0.0% (0/12)	0.0% (0/12)	0.0% (0/12)	0.0% (0/12)	10.0% (1/10)	0.8% (1/128)
Bacteria	*Campylobacter jejuni/coli*	0.0% (0/12)	33.3% (4/12)	0.0% (0/12)	0.0% (0/12)	0.0% (0/22)	0.0% (0/12)	0.0% (0/12)	0.0% (0/12)	0.0% (0/12)	0.0% (0/10)	3.1% (4/128)
	Clostridioides difficile	0.0% (0/12)	0.0% (0/12)	0.0% (0/12)	0.0% (0/12)	4.5% (1/22)	0.0% (0/12)	0.0% (0/12)	0.0% (0/12)	0.0% (0/12)	0.0% (0/10)	0.8% (1/128)
	Enteropathogenic *E. coli* (bfpA)	0.0% (0/12)	0.0% (0/12)	0.0% (0/12)	0.0% (0/12)	0.0% (0/22)	0.0% (0/12)	0.0% (0/12)	8.3% (1/12)	16.7% (2/12)	0.0% (0/10)	2.3% (3/128)
	Enteropathogenic *E. coli* (eae)	8.3% (1/12)	0.0% (0/12)	0.0% (0/12)	16.7% (2/12)	18.2% (4/22)	25.0% (3/12)	8.3% (1/12)	16.7% (2/12)	50.0% (6/12)	10.0% (1/10)	15.6% (20/128)
	Enterotoxigenic *E. coli* (LT)	0.0% (0/12)	0.0% (0/12)	0.0% (0/12)	0.0% (0/12)	0.0% (0/22)	0.0% (0/12)	0.0% (0/12)	16.7% (2/12)	0.0% (0/12)	0.0% (0/10)	1.6% (2/128)
	Enterotoxigenic *E. coli* (STp)	0.0% (0/12)	0.0% (0/12)	0.0% (0/12)	8.3% (1/12)	0.0% (0/22)	0.0% (0/12)	0.0% (0/12)	0.0% (0/12)	0.0% (0/12)	0.0% (0/10)	0.8% (1/128)
	Escherichia coli O157:H7	0.0% (0/12)	0.0% (0/12)	0.0% (0/12)	0.0% (0/12)	0.0% (0/22)	0.0% (0/12)	0.0% (0/12)	41.7% (5/12)	8.3% (1/12)	0.0% (0/10)	4.7% (6/128)
	Klebsiella pneumoniae	0.0% (0/12)	0.0% (0/12)	0.0% (0/12)	0.0% (0/12)	9.1% (2/22)	0.0% (0/12)	0.0% (0/12)	0.0% (0/12)	0.0% (0/12)	60.0% (6/10)	6.2% (8/128)
	Plesiomonas shigelloides	33.3% (4/12)	0.0% (0/12)	0.0% (0/12)	0.0% (0/12)	0.0% (0/22)	0.0% (0/12)	0.0% (0/12)	0.0% (0/12)	0.0% (0/12)	0.0% (0/10)	3.1% (4/128)
	Shiga-toxin producing *E. coli* (*stx1*)	0.0% (0/12)	16.7% (2/12)	0.0% (0/12)	8.3% (1/12)	0.0% (0/22)	8.3% (1/12)	0.0% (0/12)	16.7% (2/12)	58.3% (7/12)	0.0% (0/10)	10.2% (13/128)
	Shiga-toxin producing *E. coli* (*stx2*)	0.0% (0/12)	0.0% (0/12)	0.0% (0/12)	0.0% (0/12)	0.0% (0/22)	8.3% (1/12)	0.0% (0/12)	0.0% (0/12)	58.3% (7/12)	0.0% (0/10)	6.2% (8/128)
	Yersinia enterocolitica	0.0% (0/12)	0.0% (0/12)	0.0% (0/12)	0.0% (0/12)	0.0% (0/22)	0.0% (0/12)	0.0% (0/12)	8.3% (1/12)	0.0% (0/12)	0.0% (0/10)	0.8% (1/128)
Protozoa	*Cryptosporidium* spp.	50.0% (6/12)	25.0% (3/12)	25.0% (3/12)	0.0% (0/12)	18.2% (4/22)	0.0% (0/12)	0.0% (0/12)	8.3% (1/12)	0.0% (0/12)	0.0% (0/10)	13.3% (17/128)
	Giardia spp.	8.3% (1/12)	8.3% (1/12)	33.3% (4/12)	0.0% (0/12)	31.8% (7/22)	8.3% (1/12)	16.7% (2/12)	0.0% (0/12)	25.0% (3/12)	0.0% (0/10)	14.8% (19/128)
	Plasmodium spp.	0.0% (0/12)	50.0% (6/12)	16.7% (2/12)	0.0% (0/12)	0.0% (0/22)	8.3% (1/12)	0.0% (0/12)	8.3% (1/12)	0.0% (0/12)	0.0% (0/10)	7.8% (10/128)
Helminth	*Ascaris lumbricoides*	0.0% (0/12)	0.0% (0/12)	0.0% (0/12)	0.0% (0/12)	0.0% (0/22)	0.0% (0/12)	0.0% (0/12)	16.7% (2/12)	0.0% (0/12)	0.0% (0/10)	1.6% (2/128)
	Strongyloides stercoralis	0.0% (0/12)	8.3% (1/12)	0.0% (0/12)	0.0% (0/12)	0.0% (0/22)	0.0% (0/12)	0.0% (0/12)	0.0% (0/12)	0.0% (0/12)	0.0% (0/10)	0.8% (1/128)

Control	100.0% (12/12)	100.0% (12/12)	100.0% (12/12)	100.0% (12/12)	100.0% (22/22)	100.0% (12/12)	100.0% (12/12)	100.0% (12/12)	100.0% (12/12)	100.0% (10/10)	100.0% (128/128)	100.0% (12/12)
	Class 1 Resistance Integron (RI)	0.0% (0/12)	91.7% (11/12)	16.7% (2/12)	8.3% (1/12)	13.6% (3/22)	41.7% (5/12)	83.3% (10/12)	83.3% (10/12)	75.0% (9/12)	30.0% (3/10)	42.2% (54/128)
	Human mtDNA	8.3% (1/12)	8.3% (1/12)	8.3% (1/12)	8.3% (1/12)	4.5% (1/22)	8.3% (1/12)	8.3% (1/12)	8.3% (1/12)	0.0% (0/12)	100.0% (10/10)	14.1% (18/128)

Table includes results for all samples, including specimens later excluded from prevalence analyses due to internal-control performance, as described in the manuscript. Targets are reported as detected/not detected based on study positivity thresholds (Cq < 40 with manual thresholding). The following pathogenic targets were not detected in any samples: *Ancylostoma duodenale*, *Necator americanus*, *Schistosoma mansoni*, *Trichuris trichiura*, *Entamoeba histolytica*, enteroaggregative *E. coli*, enterotoxigenic *E. coli* (*STh*), *Helicobacter pylori*, *Salmonella enterica* serovar Typhi, *Salmonella* spp., *Shigella* spp./enteroinvasive *E. coli*, *Vibrio cholerae*, Influenza A, Norovirus GI, Rotavirus, Sapovirus, SARS-Cov2, *Candida auris*. TAC = TaqMan Array Card. Cq = quantification cycle. RT-qPCR = Reverse-Transcription Quantitative Polymerase Chain Reaction.

†This assay was designed for human feces, but may cross react with some animal feces (20)
