# Supplementary material for: Diverse enteric bacterial, viral, and parasitic pathogen genes are shed in animal feces in Indiana
Source: PLoS One. 2026 Feb 6;21(2):e0335338. doi: 10.1371/journal.pone.0335338 (PMC12880659; doi:10.1371/journal.pone.0335338)
Supplement: S7 Table — Table shows stool mass used to prepare each NaNO₃ flotation suspension, resulting suspension concentration (g mL ⁻ ¹), egg counts per 0.5 mL Mini-FLOTAC chamber, and calculated eggs-per-gram of stool (EPG). (PDF) [file pone.0335338.s008.pdf]

S7 Table. Quantitative Mini-FLOTAC microscopy summary of *Ascaris* type eggs in pig and dog fecal samples collected at 10 sites in southern Indiana, April–June 2024.

Species	Sample	Stool input Mass (g)	Microbe Species	Eggs / 0.5 mL chamber	Stool conc. (g/mL)	Eggs / g stool (EPG)
Dog	Dog 1	0.9353	none	0	0.0520	0
	Dog 2	1.6177	none	0	0.0899	0
	Dog 3	1.121	none	0	0.0623	0
	Dog 4	1.3438	none	0	0.0747	0
	Dog 5	1.0915	none	0	0.0606	0
	Dog 6	1.4283	none	0	0.0794	0
	Dog 7	0.4528	none	0	0.0252	0
	Dog 8	0.5193	none	0	0.0289	0
	Dog 9	0.7056	none	0	0.0392	0
	Dog 10	0.5586	none	0	0.0310	0
	Dog 11	0.8298	none	0	0.0461	0
	Dog 12	0.5049	none	0	0.0281	0
	Dog 13	0.4005	none	0	0.0222	0
	Dog 14	0.2913	none	0	0.0162	0
	Dog 15	0.6112	none	0	0.0340	0
	Dog 16	0.6423	none	0	0.0357	0
	Dog 17	0.5563	none	0	0.0309	0
	Dog 18	0.3508	none	0	0.0195	0
	Dog 19	0.7988	none	0	0.0444	0
	Dog 20	0.4507	none	0	0.0250	0
	Dog 21	0.572	none	0	0.0318	0
	Dog 22	0.6049	none	0	0.0336	0
Pig	Pig 1	0.3434	none	0	0.0191	0
	Pig 2	0.26	*Ascaris suum*	1014	0.0144	140400
	Pig 3	0.6901	*Ascaris suum*	2664	0.0383	138971
	Pig 4	0.8006	*Ascaris suum*	48	0.0445	2158
	Pig 5	0.5627	*Ascaris suum*	193	0.0313	12348
	Pig 6	0.7927	*Ascaris suum*	1608	0.0417	77083
	Pig 7	0.8053	*Ascaris suum*	96	0.0447	4292
	Pig 8	0.314	*Ascaris*	1236	0.0165	149580

			suum			
	Pig 9	0.6737	*Ascaris suum*	3408	0.0374	182111
	Pig 10	0.3807	*Ascaris suum*	192	0.0224	17147
	Pig 11	0.92	*Ascaris suum*	1008	0.0511	39443
	Pig 12	0.4894	*Ascaris suum*	144	0.0272	10593

Table shows stool mass used to prepare each $NaNO_3$ flotation suspension, resulting suspension concentration (g mL^{-1}), egg counts per 0.5 mL Mini-FLOTAC chamber, and calculated eggs-per-gram of stool (EPG).
