# Supplementary material for: Diverse enteric bacterial, viral, and parasitic pathogen genes are shed in animal feces in Indiana
Source: PLoS One. 2026 Feb 6;21(2):e0335338. doi: 10.1371/journal.pone.0335338 (PMC12880659; doi:10.1371/journal.pone.0335338)
Supplement: S2 Fig — Forward primer (positions 589–611) and reverse primer (638–659) are shaded in yellow and blue, respectively; the hydrolysis probe (619–636) is shaded in green. A single nucleotide mismatch at probe position 625 (boxed in red) distinguishes A. duodenale from A. ceylanicum, while three mismatches at probe positions 625, 633, and 636, as well as one mismatch as reverse primer position 644 distinguish A. duodenale from A. caninum. (PDF) [file pone.0335338.s009.pdf]

S2 Fig. In-silico alignment of *Ancylostoma duodenale* (GenBank MK271367.1) with (left) *Ancylostoma ceylanicum* (PP527745.1) and (right) *Ancylostoma caninum* (MT130933.1) sequences in the assay region. Forward primer (positions 589–611) and reverse primer (638–659) are shaded in yellow and blue, respectively; the hydrolysis probe (619–636) is shaded in green. A single nucleotide mismatch at probe position 625 (boxed in red) distinguishes *A. duodenale* from *A. ceylanicum*, while three mismatches at probe positions 625, 633, and 636, as well as one mismatch as reverse primer position 644 distinguish *A. duodenale* from *A. caninum*.

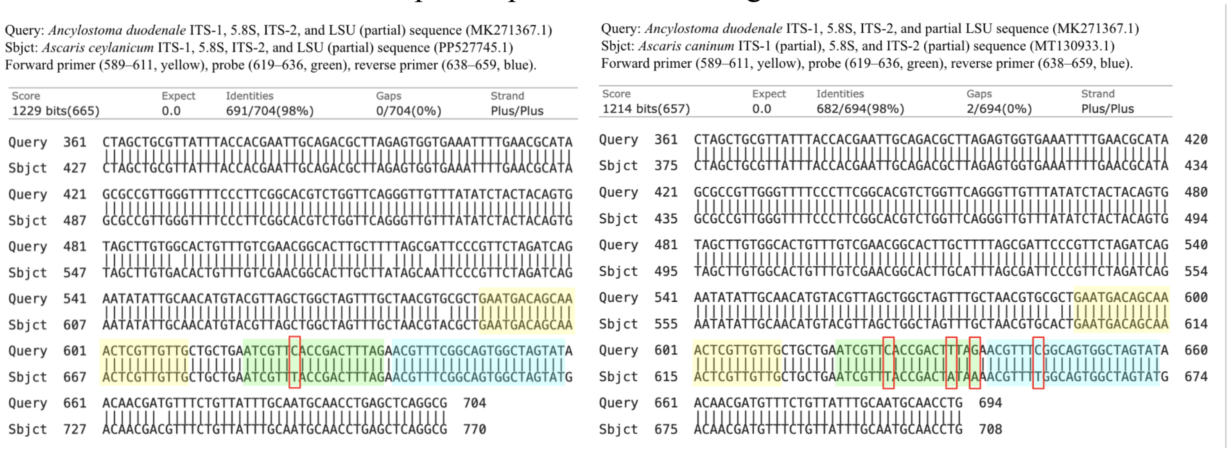

Species (accession)	Coverage	%ID	Mismatches (F / Probe / R)	Predicted amplicon (bp)	Note
Ascaris ceylanicum (PP527745.1)	100%	98.15%	0 / 1 / 0	71	Single mismatch in probe
Ascaris caninum (MT130933.1)	99%	98.27%	0 / 3 / 1	71	Four mismatches in total
