# Supplementary material for: Diverse enteric bacterial, viral, and parasitic pathogen genes are shed in animal feces in Indiana
Source: PLoS One. 2026 Feb 6;21(2):e0335338. doi: 10.1371/journal.pone.0335338 (PMC12880659; doi:10.1371/journal.pone.0335338)
Supplement: S3 Fig — The forward primer (113–137, yellow), reverse primer (225–246, blue), and probe (174–195, green) are all highlighted. A single nucleotide mismatch at probe position 178 (boxed in red) distinguishes A. suum from A. lumbricoides. (PDF) [file pone.0335338.s010.pdf]

S3 Fig. In-silico alignment of *Ascaris lumbricoides* (GenBank PP758217.1) and *Ascaris suum* (PP758228.1) sequences in the assay region. The forward primer (113–137, yellow), reverse primer (225–246, blue), and probe (174–195, green) are all highlighted. A single nucleotide mismatch at probe position 178 (boxed in red) distinguishes *A. suum* from *A. lumbricoides*.

Query: *Ascaris lumbricoides* ITS1 sequence (PP758217.1)
Sbjct: *Ascaris suum* ITS1 sequence (PP758228.1)
Forward primer (113–137, yellow), probe (174–195, green), reverse primer (225–246, blue).

Score	Expect	Identities	Gaps	Strand
1038 bits(562)	0.0	574/579(99%)	3/579(0%)	Plus/Plus

```
Query  1    GGCAAAAGTCGTAACAAGGTTTCCGTAGGTGAACCTGCGGAAGGATCATTATCGAGCAG-  59
Sbjct  1    GGCAAAAGTCGTAACAAGGTTTCCGTAGGTGAACCTGCGGAAGGATCATTATCGAGCAGA  60

Query  60   -aaaaaaaaaaGTCTCCGAACGTGCACATAAGTACTATTTGCGCGTATACGTGAGCCACA  118
Sbjct  61   AAAAAAAAAAAGTCTCCGAACGTGCACATAAGTACTATTTGCGCGTATACGTGAGCCACA  120

Query  119  TAGTAAATTGCACACAAATGTGGTGATGTAATAGCAGTCGGCGGTTTC-tttttttttGG  177
Sbjct  121  TAGTAAATTGCACACAAATGTGGTGATGTAATAGCAGTCGGCGGTTTCTTTTTTTTTTGG  180

Query  178  CGGACAATTGCATGCGATTTGCTATGTGTTGAGGGAGAATAGGTGGCATGTTGGGCTTGT  237
Sbjct  181  CCGACAATTGCATGCGATTTGCTATGTGTTGAGGGAGAATAGGTGGCATGTTGGGCTTGT  240

Query  238  TAGAAAGGCATGCCGCTAGCGCTTATTTTCCCGCTATTTCGTAACAACGGTGTCCATTTT  297
Sbjct  241  TAGAAAGGCATGCCGCTAGCGCTTATTTTCCCGCTATTTCGTAACAACGGTGTCCAATTT  300

Query  298  GGCGTCTACGCTTCACCGAGCTATCGCCTGGACCGTCGGTAGCGATGAAAGGTGGAGAGA  357
Sbjct  301  GGCGTCTACGCTTCACCGAGCTATCGCCTGGACCGTCGGTAGCGATGAAAGGTGGAGAGA  360
```

Species (accession)	Coverage	%ID	Mismatches (F / Probe / R)	Predicted amplicon (bp)	Note
Ascaris suum (PP758228.1)	100%	99%	0 / 1 / 0	135	Single mismatch in probe
