# Supplementary material for: Diverse enteric bacterial, viral, and parasitic pathogen genes are shed in animal feces in Indiana
Source: PLoS One. 2026 Feb 6;21(2):e0335338. doi: 10.1371/journal.pone.0335338 (PMC12880659; doi:10.1371/journal.pone.0335338)
Supplement: S4 Fig — The forward primer (218–242, yellow), reverse primer (272–294, blue), and probe (244–270, green) are all highlighted. A single nucleotide mismatch at probe position 225 (boxed in red) distinguishes T. trichiura from T. vulpis and T. ovis. (PDF) [file pone.0335338.s011.pdf]

S4 Fig. In-silico alignment of *Trichuris trichiura* (GenBank LC800551.1) with (left) *Trichuris vulpis* (HF586909.1) and (right) *Trichuris ovis* (HF586911.1) sequences in the assay region. The forward primer (218–242, yellow), reverse primer (272–294, blue), and probe (244–270, green) are all highlighted. A single nucleotide mismatch at probe position 225 (boxed in red) distinguishes *T. trichiura* from *T. vulpis* and *T. ovis*.

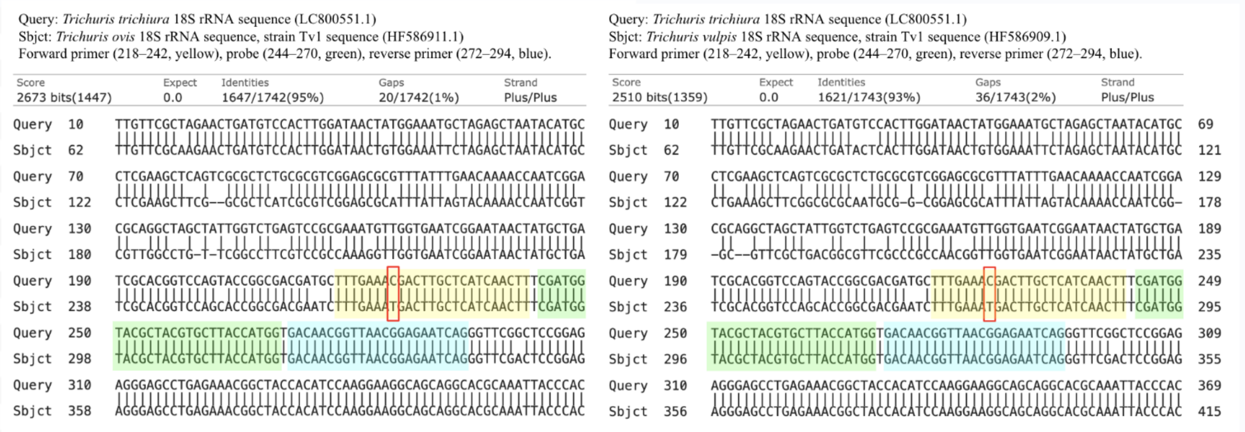

Species (accession)	Coverage	%ID	Mismatches (F / Probe / R)	Predicted amplicon (bp)	Note
Trichuris vulpis (HF586909.1)	99%	93%	1 / 0 / 0	76	Single mismatch in forward primer
Trichuris ovis (HF586911.1)	99%	94.55%	1 / 0 / 0	76	Single mismatch in forward primer
