# Supplementary material for: Diverse enteric bacterial, viral, and parasitic pathogen genes are shed in animal feces in Indiana
Source: PLoS One. 2026 Feb 6;21(2):e0335338. doi: 10.1371/journal.pone.0335338 (PMC12880659; doi:10.1371/journal.pone.0335338)
Supplement: S5 Fig — The forward primer (686–708, yellow), reverse primer (765–785, blue), and probe (716–735, green) are all highlighted. There are no mismatches distinguishing P. falciparum from P. relictum in these regions. (PDF) [file pone.0335338.s012.pdf]

S5 Fig. In-silico alignment of *Plasmodium falciparum* (GenBank MN852864.1) and *Plasmodium relictum* (PV628049.1) sequences in the assay region. The forward primer (686–708, yellow), reverse primer (765–785, blue), and probe (716–735, green) are all highlighted. There are no mismatches distinguishing *P. falciparum* from *P. relictum* in these regions.

Query: *Plasmodium falciparum* 18S rRNA (partial) sequence (MN852864.1)
Sbjct: *Plasmodium relictum* 18S rRNA (partial) sequence (PV628049.1)
Forward primer (686–708, yellow), probe (716–735, green), reverse primer (765–785, blue).

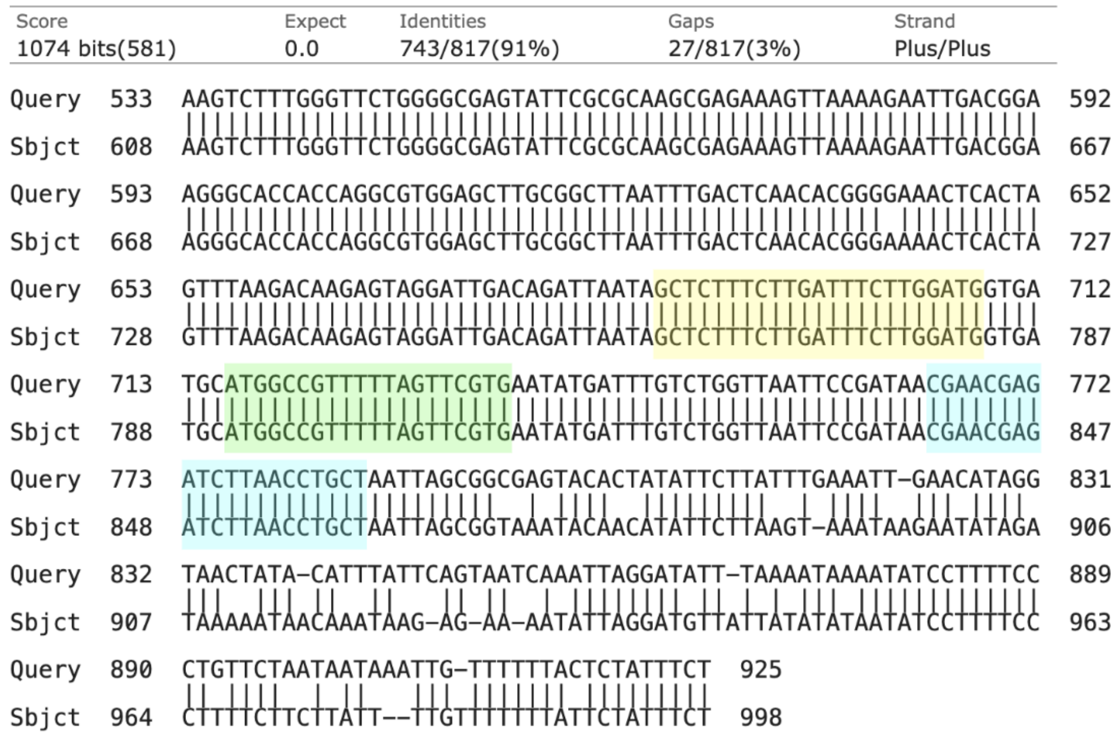

Species (accession)	Coverage	%ID	Mismatches (F / Probe / R)	Predicted amplicon (bp)	Note
Plasmodium relictum (PV628049.1)	85%	90.94%	0 / 0 / 0	100	No mismatches in primers or probe
