# Supplementary material for: Diverse enteric bacterial, viral, and parasitic pathogen genes are shed in animal feces in Indiana
Source: PLoS One. 2026 Feb 6;21(2):e0335338. doi: 10.1371/journal.pone.0335338 (PMC12880659; doi:10.1371/journal.pone.0335338)
Supplement: Attachment — Submitted filename: 3-ReviewerComment-PLOS 6Jan25.docx [file pone.0335338.s013.docx]

**Entitled:** Enteric pathogen profiles of animal feces in Indiana

Please consider the following points:

1. The study objective should be clearly stated.

2. Regarding the research methodology, aspects such as sample size require explanation. Please specify the method used to determine the number of subjects.

**Reviewer comments:**

**Minor comments:**

1.Abstract Section.

Please add topic headings such as Introduction, Methodology, Results and conclusion.

2. Introduction Section.

It is OK.

3. Methodology Section.

It is OK.

4. Results Section.

It is OK.

5. Discussion Section.

It is OK.

6. Conclusion Section.

It is OK.
